# CT images and radiotherapy treatment planning of patients with breast cancer: A dataset

**DOI:** 10.1016/j.dib.2017.06.002

**Published:** 2017-06-10

**Authors:** Mohammad Rezaei, Ahmad Mohammadbeigi, Karim Khoshgard, Abbas Haghparast

**Affiliations:** a Sleep Disorders Research Center, Kermanshah University of Medical Sciences, Kermanshah, Iran; b Students Research Committee, Faculty of Medicine, Kermanshah University of Medical Sciences, Kermanshah, Iran; c Department of Medical Physics, Faculty of Medicine, Kermanshah University of Medical Sciences, Kermanshah, Iran

**Keywords:** Bioimage dataset, Breast cancer, Radiotherapy, Lung injury

## Abstract

The data presented here were originally collected for the research project “CT-Scan processing and analysis in patient with breast cancer after radiotherapy”. Also, it reported in our study “Prediction of Lung Tissue Damage by Evaluating Clinical and Dosimetric Parameters in Breast Cancer Patients” (Hasanabdali et al., 2016) [1]. This article describes and directly links to 52 subjects referred to Mahdieh Oncology and Radiotherapy Center from February to August 2015. Treatment planning was done for delivering 50 Gy dose to PTV in 25 fractions. the lungs and heart objects were extracted from CT images along with compliance Dose plan. Dose-volume histogram (DVH) and Dose-mass histogram (DMH) extracted using CT images and dose plan matrix. Moreover, the complete clinical and dosimetric specifications of subjects is attached.

**Specifications Table**

**Table:** 

Subject area	*Biology*
More specific subject area	*Radiotherapy, cancer, psychophysiological insomnia, Respiratory*
Type of data	*Table( Excel spreadsheets), text file, m-file, mat file, image(DICOM files)*
How data was acquired	*Data were recorded using Siemens Primus linear accelerator 6 MV/15 MV Photonic and Sensation Siemens CT scan 1 slice. Also, analyzed data was acquired using Matlab software.*
Data format	*Raw data, analyzed,*
Experimental factors	*clinical and dosimetric specifications of subjects, radiation dose measured by treatment planning, CT image and respiratory information. Age, gender, height, weight, education, marriage, and body mass index were used as covariates.*
Experimental features	*the lungs and heart objects were extracted from CT images along with compliance Dose plan, DVH, DMH*
Data source location	*Samples were collected in Mahdieh Oncology and Radiotherapy Center from February to August 2015.*
Data accessibility	*The dataset is freely available at*[2]*for any academic, educational, and research purposes.*
	https://data.mendeley.com/datasets/vj83jj6fvt/draft?a=e29e5cdd-ac83-420d-b519-b55454567f61

**Value of the data**

• Raw data can be reprocessed using algorithms and other procedures in future research in the study area.
• The data presents the full-information (raw & analyzed data) for 52 subjects with breast cancer.

## 1 Experimental design, materials and methods

### 1.1 Participant

In this prospective analysis 52 patients (one patient with only external given dose) ages 34 to 71 years, (mean age: 47 years) with stages II, III breast cancers who were referred to Mahdieh radiotherapy center in Hamedan of Iran between February to August 2015 were included [1]. All patients, 25 patients with left and 26 patients with right breast involvement, which were at stages II and III (according to the American Joint Committee on Cancer (AJCC)) had undergone modified radical mastectomy (MRM) or mastectomy [3]. All patients had received prior chemotherapy with a same regimen of 8 stages one month before radiotherapy. Also, patients with smoking addiction and or had suffered from background lung and or heart diseases were previously excluded from our study. Some anatomical and dosimetric data of the patients including CLD, bridge separation (BS) and irradiated lung volumes in tangential field were obtained from the treatment plans of the patients. Summary of patients’ characteristics presented in Table 1.

**Table 1:** Summary of patients’ characteristics.

	characters	No. of patients (%)
**Age (years)**	Mean	47
	Range	34–71
**Tumor site**	Left	25(49%)
	Right	26(51%)
**surgery**	Mastectomy	27(52%)
	Modify radical mastectomy	24(48%)
**T stage**	T1	8(15%)
	T2	28(55%)
	T3	15(30%)
**N stage**	N0	7
	N1	19
	N2	22
	N3	3

### 1.2 Radiation therapy

**Fig. 1:**
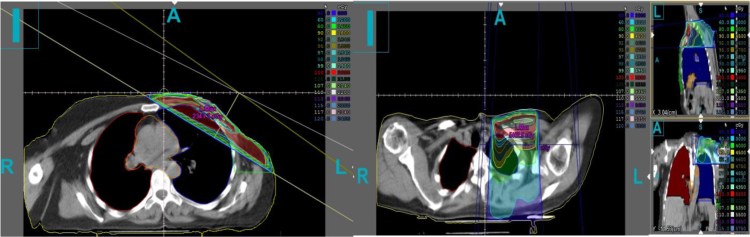
(Left) A sample breast cancer treatment plan (tangential field), (Right) supraclavicular field.

Radiotherapy of the patients was done using 6-MV and 15-MV photon beams produced by a linear accelerator of electron (Primus, Siemens, Germany). Before radiotherapy, patients were immobilized using a breast board in the supine position while both arms were expanded over the head. CT images were obtained using a single-slice CT-scan unit (Sensation, Siemens, Germany) transverse slices with thickness of 8 mm. Treatment planning of radiation therapy based on the CT-scan slices for the patients was performed using the Core-PLAN (version 3.5.0.5, Seoul C & J Co., Seoul, South Korea) treatment planning system (TPS) [4]. Dose delivery technique was single isocentric and SAD for delivering 2 Gy per day over 5 consecutive days per week. A sample of the breast cancer treatment planning is shown in Fig. 1. The breast parenchyma (chest wall in the patients with mastectomy and whole breast for modify radical mastectomies) were outlined as clinical target volume (CTV); also left and right lungs volumes were separately contoured as the organ at risk (OAR) structures. A part of ipsilateral lung volume of all patients was within the tangential radiation field. Dose calculations were carried out using equivalent tissue to air ratio (ETAR) algorithm. The absorbed dose received by the ipsilateral lung was derived from the patients’ treatment plans. CLD parameter which defined as the perpendicular distance from the posterior tangential field edge to the posterior part of the tangential field was measured on the CT images for each patient [5]. Also, the percentage of lung volume which is located in the tangential field was calculated.

The prescribed dose to the PTV was 50 Gy in 25 fractions. A number of 11 patients (6.6%) received five more fractions as a boost using 6 MV or 15 MV photon beams or 15 MeV electron beam to the tumor bed. All patients treated with tangential opposed fields, with or without an anterior supraclavicular field; however, 6 patients (6.3%) had internal mammary node (IMN) radiation field using photon or electron beams in addition to tangential and/or supraclavicular fields. Post axillary field was used in 9 patients (4.5%), too. The planning goals were to cover 95% of the target volume with 100% of the prescription dose and to keep the critical structure doses at or below known tolerance limits [5]. Tangential fields were defined in treatment planning, in which the minimum lung volume would be in radiation field while having an appropriate coverage of dose to PTV. This will lead to maximum protection of surrounding normal (lung) tissue.

### 1.3 Pulmonary function evaluation

Pulmonary function parameters were assessed using a spirometry System in the Shahid Beheshti hospital in Hamedan, Iran, according to the American Thoracic Society recommendation [6]. Parameters estimated included Dynamic or forced parameters (forced expiratory volume in 1 second [FEV 1], forced vital capacity [FVC]) were measured with spirometry. Moreover, another parameter measured that are expressed in Table 2.

**Table 2:** Pulmonary function parameters for a patient as example: Parameter values and reference values and their percentage are also expressed.

	value3	%Ref	Ref	Unit
FVC	2.77	127	2.18	L
FEV1	1.85	102	1.81	L
FEV1/IVC	–	–	78	%
FEV1/FVC	67	86	78	%
PEF	4.57	86	5.32	L/S
MEF25	3.01	61	4.92	L/S
MEF50	1.53	46	3.34	L/S
MEF75	0.27	23	1.2	L/S
MEF25-75	0.96	35	2.78	L/S
Aex	4.7	–	–	L^⁎^L/s

All of these pulmonary function tests were accomplished before and after completion of Radiotherapy, 3 and 6 months afterwards and were examined by a pulmonologist. The Common Toxicity Criteria for Adverse Events (version 4.03) of the National Cancer Institute [7] were used for the definition of Pneumonitis. Values were coordinated by sex, age, weight and height and expressed as a percentage of predicted reference values [8]. Total spirometry data for 52 patient packaged in *Spirometry.mat*.

### 1.4 Data processing

**Fig. 2:**
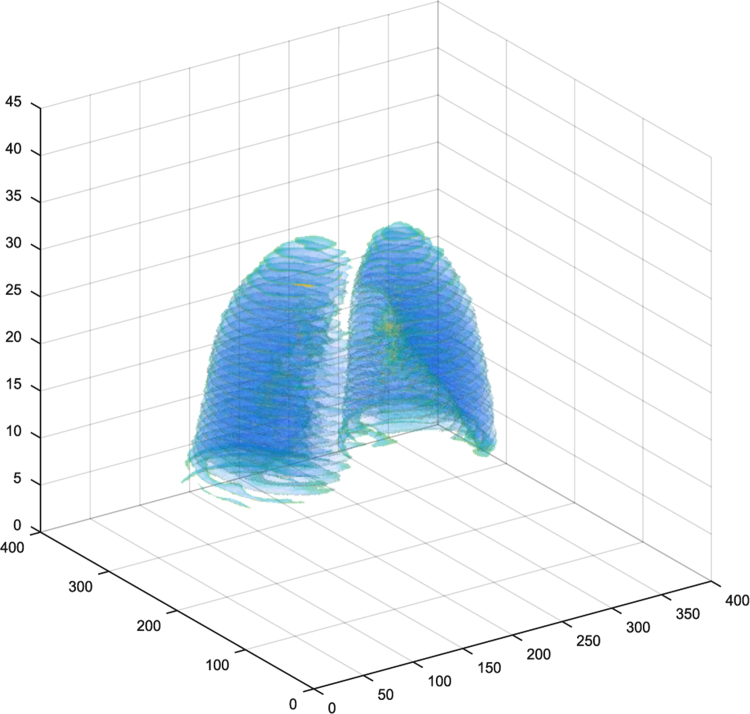
Lungs in 3D extracted using “*Object3D.m”*.

Data including CT images and spirometry information have only been collected, but other data including organs segmentation, dose-volume histogram (DVH), volume -dose histogram (VDH) and dose-mass histogram (DMH), are processed. A program, “*AcquisitionDicom.m”*, is responsible for Individually segmentation and extracting organs from CT images. Extracted organs is including the lungs, the heart, Part of body is known as the External and clinical target volume (CTv). Classified information is such that can be display one or more organs in 3D for each patient. A program, “*Object3D.m”*, is responsible for this task. Fig. 2 shows lungs in 3D.

**Fig. 3:**
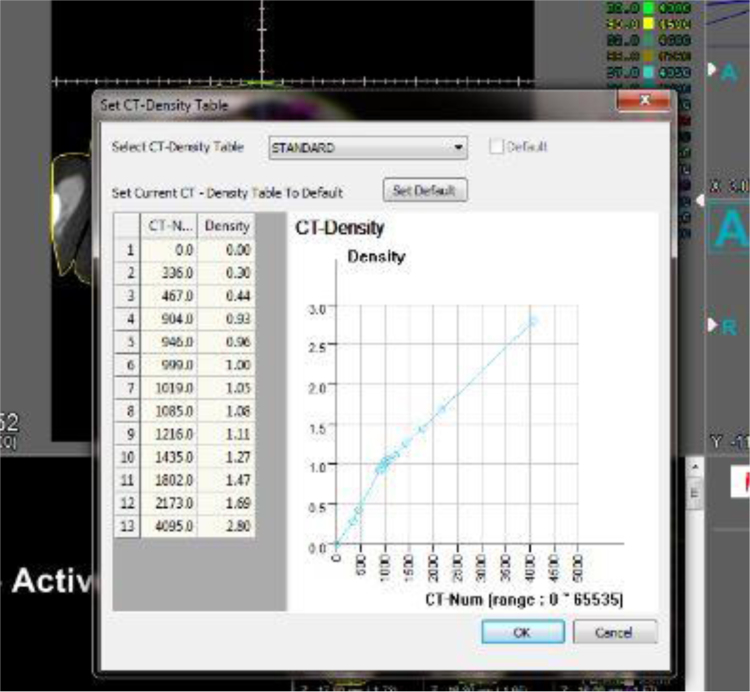
Mentioned transform chart for transformation CT-Number to density in CorePLAN software.

DVH and VDH data extraction is also done by *“DVH_Calc.m”* and *“VDH_Calc.m”* based on CT images and dose matrix. For extracting DMH data, in firstly, density based on CT number and then mass of voxels is calculated. A program, *“DMH_Calc.m”* and a sub program *“ToMass.m”* are containing above steps. To convert the intensity values of each voxel in the CT images to mass is used transform chart providing by CorePLAN software. In Fig. 3 shown mentioned transform chart.
