# Supplementary material for: CT images and radiotherapy treatment planning of patients with breast cancer: A dataset
Source: Data Brief. 2017 Jun 10;13:390–5. doi: 10.1016/j.dib.2017.06.002 (PMC5480817; doi:10.1016/j.dib.2017.06.002)
Supplement: Supplementary file 1 — Supplementary material [file mmc1.docx]

**I do not have any conflict of interest**
